# Need-supportive coaching and engagement in Chinese high school football players: a serial mediation model

**DOI:** 10.3389/fpsyg.2024.1466985

**Published:** 2025-01-10

**Authors:** Chuantong Jiang, Norsilawati Abdul Razak, Nelfianty Mohd Rasyid

**Affiliations:** ^1^Faculty of Physical Education and Health, Zhaoqing University, Zhaoqing, China; ^2^Faculty of Sports Science and Coaching, Sultan Idris Education University, Tanjong Malim, Malaysia

**Keywords:** need-supportive coaching behavior, subjective task value, task orientation, engagement, football players

## Abstract

**Introduction:**

This study aimed to investigate the relationship between need-supportive coaching behavior, subjective task value, goal orientation, and engagement among Chinese high school football players and propose four research hypotheses better to understand the determinants and mechanisms of athlete engagement.

**Methods:**

Participants were 385 Chinese high school football players (228 boys; 157 girls; Mage = 16.07 years; SD = 1.15; range = 14–19 years) on 20 teams from 45 high schools using a proportional stratified random sampling method. Participants filled out previously validated questionnaires, including the Interpersonal Behaviors Questionnaire (IBQ), Subjective Task Value (STV), The Task and Ego Orientations Questionnaire (TEOSQ), and Athlete Engagement Questionnaire (AEQ). To address the hypothesis testing, this study utilized structural equation modeling (SEM) to analyze the proposed multiple mediation model.

**Results:**

The research results indicated that need-supportive coaching behavior had a significant direct (*β* = 0.121, *Z* = 3.667, *p* < 0.001) and indirect (*β* = 0.209, *Z* = 5.500, *p* < 0.001) effect on athlete engagement. Moreover, need-supportive coaching behavior positively predicted athlete engagement through subjective task value (*β* = 0.128, *Z* = 4.000, *p* < 0.001) and task orientation (*β* = 0.053, *Z* = 3.118, *p* < 0.001). Also, subjective task value and task orientation sequentially mediated the relationship between need-supportive coaching behavior and athlete engagement (*β* = 0.028, *Z* = 3.500, *p* < 0.001).

**Discussion:**

The findings demonstrate that need-supportive coaching behavior, subjective task value, and task orientation are important factors in promoting athlete engagement. The study implies that encouraging and guiding coaches to adopt a need-supportive coaching style is an effective motivational strategy, which can not only directly predict athlete engagement, but also indirectly influence athlete engagement through subjective task value and task orientation.

## 1 Introduction

Throughout the years, the Chinese government has placed a great emphasis on the development of football, providing it with strong, sustained support. Since 2015, the Chinese government has formulated a Mid – and Long-term Development Plan for Chinese football (2016–2050), expecting to encourage youth involvement in football through the campus football model, to improve the popularization and competitive level of football (Wang and Fan, 2018). However, the reality is that football is far less popular in Chinese schools than many other sports, such as basketball, table tennis, swimming, and badminton (Chai and Lin, 2018). In addition, it is found that teenagers, especially high school students, can maintain high enthusiasm in the early stage of participating in football, but such enthusiasm is not lasting, and a considerable number of high school students have the phenomenon of enthusiasm fading or even premature disengagement (Wu, 2016). Therefore, the problem to be addressed was the lack of engagement among Chinese high school football players, which might lead to their premature disengagement from the sport. This is not conducive to the national strategy of improving the popularization and competitive level of football in China through the campus football model. It’s worth noting that the loss of youth sports commitment is a noticeable issue in many countries (Crane and Temple, 2015), and thus, the problem addressed in the context of China can be linked to a broader, global motivation and sports participation issue.

In sports, athlete engagement is an enduring experience characterized by positive cognitive and emotional states, such as confidence, dedication, enthusiasm, and vigor (Hodge et al., 2009). Research on coaching behavior, together with the accumulation of practitioner wisdom, has shown that athlete’s motivation and engagement can be significantly influenced by coaches who employ various strategies to inspire and encourage them (Jackson-Kersey and Spray, 2016). Gaining knowledge about how coaches motivate athlete engagement can provides a fuller comprehension of the intricacies of human behavior in sports, and establish a framework for promoting positive sports experiences, as well as provide empirical evidence and motivational strategies for coaches to create a positive coaching climate and intervention on athletes’ motivation to participate. These can also provide practical implications for coaches or football organizations. For example, the results can guide coaches to adopt need-supportive behaviors so as to enhance athletes’ motivation and engagement. Besides, football organizations can make use of these insights to design more effective policies to maintain youth participation. By taking both theoretical and practical aspects into account, this study endeavors to make a meaningful contribution to the discussions related to youth football participation.

To date, researchers have discussed the antecedents of athlete engagement from different perspectives. According to Self-Determination Theory (SDT), need-supportive coaching behavior, as social and contextual factors, can affect athletes’ autonomous motivation and engagement (Rottensteiner et al., 2015; Aelterman et al., 2017; Berntsen and Kristiansen, 2019; Reynders et al., 2019; De Muynck et al., 2020). Meanwhile, from different theoretical perspectives, some researchers have proposed that subjective task value, and task orientation as personal factors are the key internal motivational processes to initiate, continue, and sustain engagement in sports (Olivier et al., 2020; Jakobsen, 2022). However, previous research has overlooked the interaction between social environments and personal factors on athlete engagement. These factors do not have a singular impact, but have a comprehensive impact on athlete engagement. Specifically, although several research has investigated social context and individual factors that influence athlete engagement (Lower et al., 2014; Reynders et al., 2019; De Muynck et al., 2020), little research has been conducted to investigate whether social-contextual factors (i.e., need-supportive coaching behavior) indirectly affect on athlete engagement through personal factors (i.e., subjective task value, and task orientation). In conjunction to this issue, more multi-dimensional studies need to investigate the psychological factors influencing athlete engagement in football.

According to the reciprocal interaction model proposed by Bandura (1986) in Social Cognitive Theory (SCT), behavioral, environmental, and personal processes influence each other reciprocally (Schunk and DiBenedetto, 2020). Under the guidance of SCT, this study attempts to clarify the field of athlete engagement by developing a contextual model that logically integrates social context, personal factors, and engagement behavior, to better comprehend the determinants and influencing mechanisms of athlete engagement. Among them, need-supportive coaching behavior is the social context factor that affects the athlete’s engagement, while the personal factor is the subjective task value and task orientation. Thus, this investigation aims to explore the relationship between need-supportive coaching behavior, subjective task value, task orientation, and athlete engagement.

## 2 Literature review and hypothesis development

### 2.1 Need-supportive coaching behavior and athlete engagement

Research in sports science has extensively explored the influence of coaches’ interpersonal behavior, particularly focusing on the concept of need-supportive coaching behavior rooted in SDT (Berntsen and Kristiansen, 2019; Ryan and Deci, 2018). This theoretical framework emphasizes the significance of coaches fostering athletes’ autonomy, competence, and relatedness to enhance motivation and participation. Studies have consistently shown that need-supportive coaching behavior positively correlates with athlete engagement (De Muynck et al., 2020; Mellano et al., 2020; Gao et al., 2021). Specifically, autonomy support provided by coaches has been seen as an important part of promoting athlete engagement, as athletes feel empowered to make choices and decisions regarding their training and performance (Pedro and Martins, 2017; Jowett et al., 2020). Additionally, coaches’ competence support, manifested through feedback and guidance, significantly contributes to athletes’ sense of efficacy and motivation, thereby enhancing their engagement with the sport (Curran et al., 2016; de et al., 2020; Podlog et al., 2015). Moreover, studies have highlighted the importance of coaches’ relatedness support in fostering positive interpersonal relationships within the sports environment, which in turn predicts higher levels of athlete engagement (Gonzalez-Peno et al., 2021; Guo et al., 2021; McGee and DeFreese, 2019).

Moreover, in the field of physical education, several studies have also investigated the influence of need-supportive teaching behavior on student engagement. Findings suggest that similar to sports coaching, need-supportive teaching behavior in educational settings positively relates to student engagement and negatively to disengagement (van et al., 2016). Competence support provided by teachers has been found to be an important determinant of student engagement, and students are likely to engage more actively in learning activities when they feel competent in their abilities (Gonzalez-Peno et al., 2021). Moreover, studies have delved into the mediating mechanisms between need-supportive teaching behavior and student engagement, revealing that need-supportive educational practices enhance engagement through the satisfaction of students’ basic needs and internal motivation (Leo et al., 2020). Overall, these results highlight the pivotal role of need support in both sports coaching and educational contexts, highlighting its positive association with athlete engagement, respectively. Therefore, in light of these evidences, we put forward the following hypothesis:

*Hypothesis 1*: Need-supportive coaching behavior is positively associated with athlete engagement.

### 2.2 Subjective task value and task orientation as the underlying mechanisms

Subjective task value refers to the perceived worth of participating in a specific task or activity, encompassing factors like attainment, intrinsic, utility value, and cost (Wigfield and Eccles, 2020). Based on the Expectation-Value Theory (EVT), subjective task value may be affected by attitudes toward socializers (Eccles et al., 1983), with coaches playing a pivotal role in shaping athletes’ beliefs about their abilities and task value. SDT further emphasizes that individuals tend to display intrinsic motivation when their psychological needs are satisfied by coaching behavior (Ryan and Deci, 2017). Recent studies underscore the significance of autonomy, competence, and relatedness support in influencing subjective task value, with teacher competency and autonomy support significantly impacting learners’ motivation and academic performance (Zhang et al., 2012; Olivier et al., 2020; Shang et al., 2022). Additionally, a caring climate perceived in primary school physical education positively influences their task value perception (Jung, 2019). Structured curricula also play vital roles in shaping task value perceptions (Pang, 2014). As a result, previous findings support the association between need-supportive coaching behavior and subjective task value.

In turn, EVT further explains the role of subjective task value on physical activity participation and positive intent (Kirk, 2019; Jaf et al., 2021). Individual perceptions of task value predict engagement and achievement across various activities (Olivier et al., 2020), including sports engagement. Studies indicate that individuals tend to participate in activities that seem both achievable and personally significant, whether in sports or academic settings (Gao, 2009; Wigfield and Eccles, 2020; Zhang et al., 2012). Research has linked students’ concentration, persistence, and effort in physical education classes to their perceptions of the task’s value (Shang et al., 2022), with consistently high task value correlating with greater engagement in sports during adolescence (Wang et al., 2017). Similarly, subjective task value has been associated with future sports engagement and educational aspirations among student-athletes (Selänne et al., 2016). Furthermore, studies have explored how intrinsic, attainment, and utility value influence engagement in physical activities, emphasizing their predictive power in determining students’ involvement in physical education (Chen and Chen, 2012; Ding et al., 2013; Yli-Piipari and Kokkonen, 2014). As a result, these findings support the role of subjective task values in driving sports engagement. In summary, it can be argued that a need-supportive climate created by coaches can enhance athletes’ task value perception, which in turn further motivates their participation and achievement. Accordingly, drawing from these findings, we put forth the following hypothesis:

*Hypothesis 2*: Subjective task value mediates the relationship between need-supportive coaching behavior and athlete engagement.

Task orientation refers to an individual’s focus on improving oneself, developing skills, and mastering tasks instead of comparing with others or solely aiming to win (Rottensteiner et al., 2015). The literature reviewed suggests a significant relationship between need-supportive coaching behavior and athletes’ task orientation. For instance, according to Diseth and Samdal (2014), autonomy support was positively related to mastery, performance-approach, and performance-avoidance, indicating a direct influence of coaching behavior on goal orientation. A person’s autonomy support has a significant effect on goal orientation, as noted by Shih (2013). D’Astous et al. (2020) discovered that athletes’ perception of competence predicts task-approach goals, suggesting that coaches’ support in fostering competence can influence athletes’ task orientation. Furthermore, Iwasaki and Fry (2016) demonstrated that athletes’ perception of a caring and task-involving atmosphere created by coaches predicted their task orientation. Additionally, Nicholls et al. (2017) revealed that coach-athlete relationships were associated with mastery goals, implying the importance of supportive coaching behaviors in cultivating task-oriented athletes. These findings collectively emphasize the significant impact of need-supportive coaching behavior on athletes’ task orientation, highlighting the crucial role coaches play in shaping athletes’ achievement goals.

Moreover, research in Achievement Goal Theory (AGT) and its related advancements indicate that task orientation significantly predicts athlete engagement. For example, Atkins et al. (2015) revealed that task orientation positively impacts athletes’ enjoyment and intention to continue sports, particularly when fostered by a supportive coaching climate. Similarly, Iwasaki and Fry (2016) found that task orientation among high school female football players predicts heightened mindfulness engagement, leading to more focused participation and positive outcomes. Sun et al. (2015) emphasized the significance of task orientation in improving adolescent students’ inclination toward sports activities when coupled with autonomy motivation support. Additionally, D’Astous et al. (2020) highlighted the role that task-based goals play in predicting the return of injured college athletes to sports, further underlining the significance of task orientation in sustaining athlete engagement. In the context of physical education, Lodewyk (2019) observed a significant correlation between task orientation and physical education participation, indicating its relevance in educational settings. Ruiz-González et al. (2015) echoed this sentiment, revealing a positive association between task orientation, autonomous motivation, enjoyment, and reduced boredom in physical education classes. Overall research shows that task orientation contributes to positive athlete experiences and sustained participation in sports. Based on these findings, we propose a third hypothesis:

*Hypothesis 3*: Task orientation mediates the relationship between need-supportive coaching behavior and athlete engagement.

To our knowledge, several studies have highlighted a correlation between an individual’s perception of the value associated with a specific task and their achievement goals. According to EVT, an individual’s task value is believed to directly influence their achievement goals (Wigfield and Eccles, 2020). For example, individuals who view tasks as valuable, interesting, and important tend to employ strategies that reflect goal engagement in future activities, indicating that a higher subjective task value leads to greater utilization of task goal orientation (Von Keyserlingk et al., 2022). Moreover, Wentzel et al. (2017) discovered that the association between social support and mastery orientation can be explained by subjective task value, underscoring the predictive role of subjective task value in task orientation. In summary, based on the aforementioned theories and studies, it appears that need-supportive coaching behavior could potentially predict athlete engagement through a sequential pathway involving subjective task value and task orientation. In other words, when soccer players perceive need support from coaches, they will recognize the value of task activities and adopt a task goal orientation, thereby enhancing their engagement in sports. Consequently, we propose that,

*Hypothesis 4*: Subjective task value and task orientation serially mediate the relationship between need-supportive coaching behavior and athlete engagement.

### 2.3 The present study

**Figure 1:**
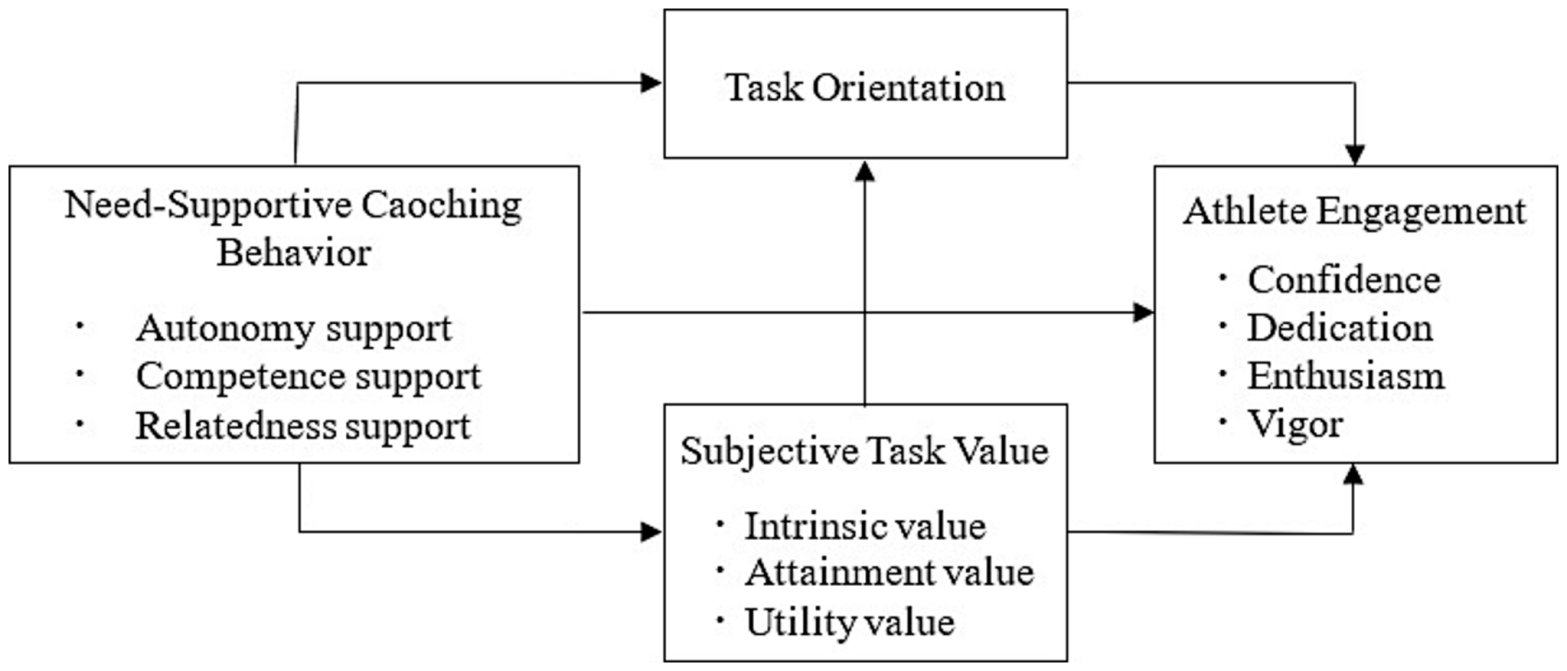
Proposed serial mediation model.

This study integrates the core concepts of Self-Determination Theory, Expectancy-Value Theory and Achievement Goal Theory, and puts forward a contextualized and generally applicable engagement model. This multi-dimensional research approach emphasizes the interaction between coaching behaviors and athletes’ internal motivational factors, making innovative theoretical contributions to the field of sports psychology. However, these influencing factors have not yet been integrated into a study to further validate Chinese high school football players. As a result, we propose a sequential mediation model (see Figure 1) that examines the relationship between need-supportive coaching behavior and athlete engagement, and the possible mediator mechanism. Based on relevant theories and research progress, we hypothesize that need-supportive coaching behavior positively predicts athlete engagement (H1), subjective task value (H2) and task orientation (H3) respectively mediate the relationship between need-supportive coaching behavior and athlete engagement, and subjective task value and task orientation serve as a serial mediator (H4). These results will contribute to the current literature and the development of campus football in China by expanding our understanding of the mechanisms linking need-supportive coaching behavior and athlete engagement.

## 3 Materials and methods

### 3.1 Participants

The participants were high school football players from Zhaoqing, China, covering both academic and vocational high schools. Using proportional stratified random sampling, athletes were selected from 45 boys’ teams and 27 girls’ teams representing 36 academic high schools and 9 vocational high schools. Based on the sample size recommended by Hair et al. (2018), 13 boys’ teams and 7 girls’ teams (ratio 45:27) were first randomly selected by gender, followed by 10 academic high school teams and 3 vocational high school teams (ratio 36:9) randomly selected by school type. There were 385 high school football players recruited for this study, including 228 boys and 157 girls, whose average age was 16.07 years (SD = 1.15). Power analysis using the R program indicated that the sample size of 385 in this study had a statistical power of 1, thus this study has sufficient confidence in the correctness of the results (MacCallum et al., 1996). According to the results of an independent sample *t*-test, there was no significant difference in any variables between football players of different genders and school types (*p* > 0.05). Additionally, all participants had engaged in at least one season of training and competition, filling different roles on the football field: 10% (39) were goalkeepers, 34% (131) were defenders, 25% (98) were midfielders, and 30% (117) were forwards.

### 3.2 Instruments

#### 3.2.1 The interpersonal behaviors questionnaire (IBQ) in sport

The subscale of the IBQ in Sport, designed by Rocchi et al. (2016), was employed to evaluate athletes’ perceptions of need-supportive coaching behavior. This scale comprises 12 observed items across three dimensions: autonomy support (e.g., “My coach supports my decisions”), competence support (e.g., “My coach provides valuable feedback”), and relatedness support (e.g., “My coach relates to me”), with 4 items in each dimension. Athletes rated each question from 1 to 7, with 1 meaning “do not agree at all” and 7 meaning “completely agree.” This study found that the sub-questionnaire had good internal consistency reliability (*α* = 0.85). Moreover, the confirmatory factor analysis (CFA) results demonstrated adequate model fit, with *χ*^2^ = 123.834, *χ*^2^/df = 2.428, CFI = 0.947, TLI = 0.976, RMSEA = 0.061, and SRMR = 0.038.

#### 3.2.2 The task and ego orientation questionnaire (TEOSQ)

This questionnaire, constructed by Nicholls (1984) and then adapted by Duda and Nicholls (1992) for application in sports, and further adapted into a Chinese version by Chinese researchers Chen and Si (1999), was designed to assess the goal orientation of athletes. The task orientation subscale includes 7 items that measure how hard athletes work to learn and master new skills, such as “I learn a new skill by trying hard.” The participants rated each item from 1 to 5, where 1 signified strong disagreements and 5 signified strong agreements. The questionnaire used in this study demonstrated high reliability (*α* = 0.92). Moreover, the CFA results demonstrated adequate model fit, *χ*^2^ = 189.171, *χ*^2^/df = 2.956, CFI = 0.963, TLI = 0.954, RMSEA = 0.072, SRMR = 0.044.

#### 3.2.3 Subjective task value (STV) scale

The STV questionnaire, developed by Eccles et al. (1983) and further applied by Stuart (2003) in the field of sports, was used to assess athletes’ subjective task values. In the STV inventory, there are three separate scales (i.e., utility, attainment, and intrinsic value), each with 2 items, such as “In general, I find playing soccer…”. Scales are rated from 1 to 7 in order to measure the subjective value of three two-item subscales. The higher the score, the more important, interesting or useful. The questionnaire demonstrated a high level of internal consistency (*α* = 0.90). Meanwhile, the CFA results showed an adequate model fit, *χ*^2^ = 12.960, *χ*^2^/df = 2.160, CFI = 0.996, TLI = 0.990, RMSEA = 0.055, SRMR = 0.011.

#### 3.2.4 Athlete engagement questionnaire (AEQ)

Athlete engagement was assessed using the AEQ constructed by Lonsdale et al. (2007). This questionnaire contains the following sub-dimensions: confidence, dedication, enthusiasm, and vigor. Sample items include “I am confident in my abilities,” “I am devoted to my sport,” “I enjoy my sport,” and “I feel mentally alert when I participate in my sport.” Participants rated these statements from 1 to 5, indicating their frequency of experiencing each feeling over the past 3 months. For these subscales, Cronbach’s alpha values were between 0.88 and 0.90, which indicates a high level of reliability. Furthermore, the CFA for the second-order model yielded a reasonable model fit, *χ*^2^ = 255.660, *χ*^2^/df = 2.557, CFI = 0.965, TLI = 0.958, RMSEA = 0.064, SRMR = 0.042.

### 3.3 Procedure

The research process included the following steps. First, the subscale of the IBQ in Sport, STV scale, and AEQ were translated into Chinese through the back-translation method for use by the participants in this study (Brislin, 1970). Next, 30 high school football players who were not selected for the formal survey were randomly chosen to conduct the pilot study, ensuring the content validity and cultural adaptation of the translated questionnaires. Finally, data were collected through structured questionnaires following standard procedures and ethical considerations. The UPSI Research Ethics Committee approved this study, and the local education bureau, principals, and coaches authorized the questionnaire survey. In all cases, participants and their parents or guardians were informed of the study’s objectives, anonymity, and confidentiality procedures. During the study, athletes voluntarily participated and signed informed consent forms, which were co-signed by both the athletes and their parents or guardians.

### 3.4 Data analysis

Data analysis was conducted using the SPSS 25 and AMOS 24 software programs, including descriptive statistics and structural equation modeling (SEM). First, necessary data screening was performed on the collected data, including missing value imputation, outlier detection, and normality assessment. Next, descriptive and correlation analyses were conducted on the demographic variables and related concepts of this study. Then, according to Anderson and Gerbing (1988), a two-step strategy, the reliability and validity of measurement models were tested to validate the structural model for the study. Finally, to verify the proposed research hypotheses, a multivariate serial mediation model was established to assess the direct and indirect effects of need-supportive coaching behavior on athlete engagement.

In the SEM analysis process, parameter estimates for the measurement and structural models were performed using the maximum likelihood method. The model was tested using several fit indices (Jackson et al., 2009; Kline, 2015; Hair et al., 2018), including chi-square (*χ*^2^), normed chi-square (*χ*^2^/df), comparative fit index (CFI), Tucker–Lewis index (TLI), root mean square error of approximation (RMSEA), and standardized root mean square residual (SRMR). According to Hair et al. (2018), the normed chi-square value less than 3, CFI and TLI values at or above 0.95, RMSEA value below 0.08, and SRMR value below 0.05 indicate an adequate fit between the hypothesized model and the observed data. Furthermore, considering that the product of the unstandardized path coefficients for the mediating variables does not meet the assumption of normal distribution, this study employed bootstrapping, a non-parametric resampling process, to assess the mediation effects (MacKinnon, 2008).

## 4 Results

To ensure compliance with SEM assumptions, this study thoroughly screened the collected data, which involved checking sample size adequacy, evaluating missing data, detecting outliers, and assessing normality. Specifically, this study obtained data from 385 cases, meeting the sample size requirements for SEM analysis. Although there were missing data among 19 observed variables, the missing rate was less than 1%, and the analysis indicated that these missing values were random and irregular. Subsequently, missing data were imputed using the built-in Bayesian methods in Amos (Buhi et al., 2008). By examining the frequency distributions of histograms, box plots, and standardized z-scores, we detected and removed six potential univariate outliers, ensuring that all item *z*-scores were within an absolute value of 4. The Mahalanobis *D*^2^ measure showed no potential multivariate outliers in this study (Kline, 2015). Based on the normality assessment presented in Table 1, skewness was between −1.098 and −0.479, and kurtosis were between −0.557 and 0.472, satisfying the normality assessment criteria (Hair et al., 2018). In summary, after data screening, 379 sample data were retained, which met the requirements for SEM analysis.

**Table 1:** Descriptive analysis, normality, correlation, reliability, and discriminant validity (*N* = 379).

	1	2	3	4
1. Need-supportive coaching behavior	**0.822**			
2. Task orientation	0.328**	**0.792**		
3. Subjective task value	0.286**	0.453**	**0.865**	
4. Athlete engagement	0.432**	0.642**	0.780**	**0.837**
Mean	5.677	4.272	5.974	4.327
Standard deviation	0.954	0.654	1.001	0.629
Skewness	−0.479	−0.598	−1.098	−0.710
Kurtosis	−0.241	−0.557	0.472	−0.460
Cronbach’s *α*	0.845	0.920	0.899	0.903

A diagonal element in bold represents the square root of AVE. The elements below the diagonal in the matrix are the Pearson correlation coefficients between the latent constructs; **p < 0.01.

The next step was to characterize the samples and perform bivariate correlation analyses. As shown in Table 1, the average score of athletes’ perception of need-supportive coaching behavior was 5.677 (SD = 0.954), the average score of task goal orientation was 4.272 (SD = 0.654), the average score of subjective task value was 5.974 (SD = 1.001), and the average score of athlete engagement was 4.327 (SD = 0.629). These results indicated that participants believed their coaches exhibited high levels of need-supportive coaching behavior, and respondents demonstrated high levels of task goal orientation, subjective task value, and sports engagement. Additionally, all bivariate correlation estimates were statistically significant (*p* < 0.01). As shown in Table 1, subjective task value and engagement (*r* = 0.780), task orientation and engagement (*r* = 0.642) showed a strong correlation. Task orientation and subjective task value (*r* = 0.453), need-supportive coaching behavior and athlete engagement (*r* = 0.371), and need-supportive coaching behavior and task orientation (*r* = 0.328) were moderately correlated. Need-supportive coaching behavior was weakly correlated with subjective task value (*r* = 0.286). Overall, based on Grewal et al. (2004), these results indicate that there were no unrelated variables or multicollinearity issues among the variables.

According to Anderson and Gerbing (1988), the CFA was conducted on the four measurement models and the overall measurement model prior to the analysis of structural models. As shown in Table 2, all standardized factor loadings exceeded the 0.7 standard and were statistically significant (*p* < 0.001), so all items were retained (Hair et al., 2018). Furthermore, the overall measurement model results indicated a good data fit, *χ*^2^(164) = 299.770, *p* < 0.001, *χ*^2^/df = 2.653, CFI = 0.959, TLI = 0.950, RMSEA = 0.066, SRMR = 0.034. Although the *p*-value was significant, this issue may be attributable to the high sample size (Marsh et al., 2004). The AVE estimates were 0.675, 0.628, 0.749, and 0.701, with item reliability ranging from 0.497 to 0.810, all generally meeting the 0.5 empirical rule. Composite reliabilities were 0.862, 0.922, 0.899, and 0.903, all exceeding the 0.7 standard. Overall, these results demonstrate the convergent validity of the measurement models. On the other hand, as shown in Table 1, the arithmetic square roots of AVE exceeded the absolute correlation coefficients, confirming differences and discriminant validity among the variables (Fornell and Larcker, 1981).

**Table 2:** Confirmatory factor analysis (CFA) results for the measurement model.

Construct	Item	Parameter significance estimation				Convergent validity			
		Unstd.	S.E.	*t*-value	*P*	Std.	SMC	CR	AVE
Need-supportive coaching behavior	AS	1.000				0.805	0.648	0.862	0.675
	CS	0.998	0.059	16.974	***	0.879	0.773		
	RS	1.262	0.080	15.737	***	0.778	0.605		
Task orientation	TO1	1.000				0.754	0.569	0.922	0.628
	TO2	0.869	0.062	14.011	***	0.705	0.497		
	TO3	1.043	0.064	16.253	***	0.804	0.646		
	TO4	0.967	0.058	16.580	***	0.818	0.669		
	TO5	1.011	0.058	17.507	***	0.857	0.734		
	TO6	0.902	0.063	14.371	***	0.721	0.520		
	TO7	1.086	0.061	17.879	***	0.873	0.762		
Subjective task value	AV					0.845	0.714	0.899	0.749
	IV	1.113	0.051	21.775	***	0.900	0.810		
	UV	1.056	0.052	20.196	***	0.850	0.723		
Athlete engagement	CON	1.000				0.836	0.699	0.903	0.701
	DED	1.012	0.052	19.361	***	0.830	0.689		
	ENTH	0.920	0.048	19.310	***	0.828	0.686		
	VIG	0.943	0.047	20.177	***	0.853	0.728		

*N* = 379. Unstd, unstandardized factor loading; S.E., standard error; *t*-value = critical ratios; ***p < 0.001; Std, standardized factor loading; SMC, item reliability; CR, composite reliability; AVE, average variance extracted.

**Figure 2:**
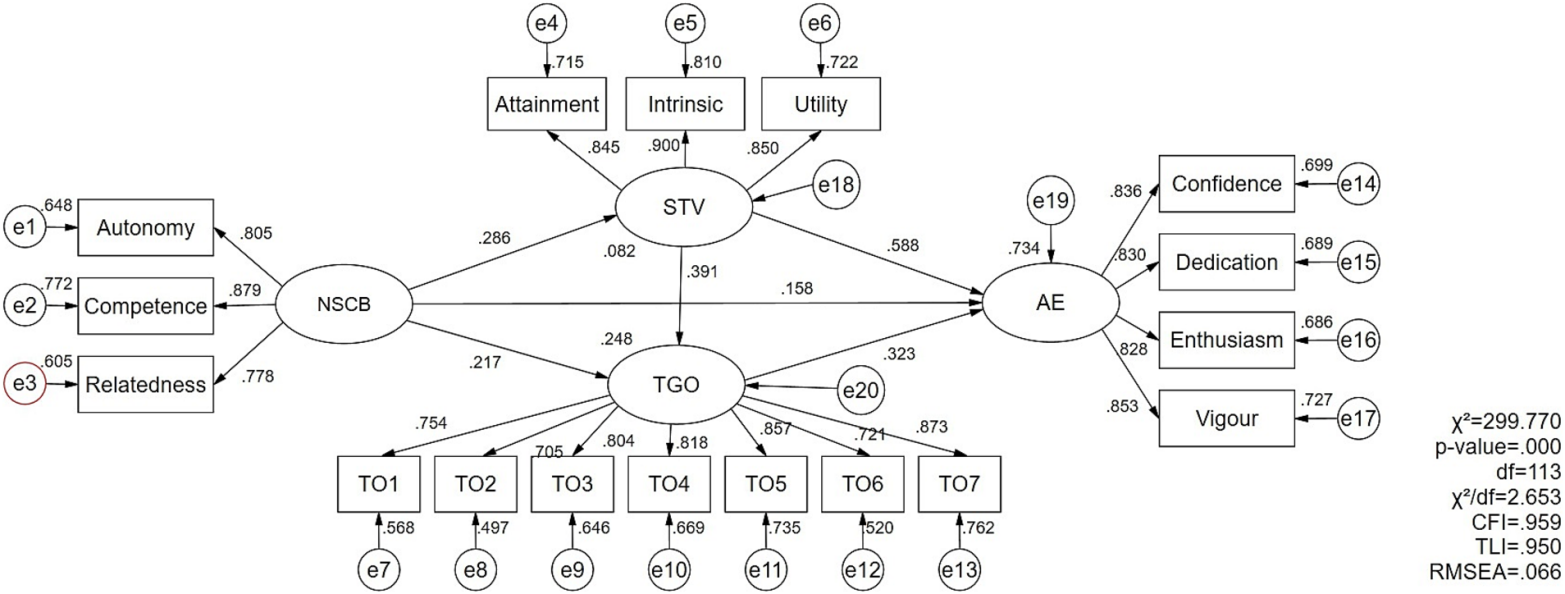
Results of structural model analysis with standardized coefficients and *R^2^*.

As shown in Figure 2, the structural model analysis examined the path coefficients between variables and the proportion of variance explained by exogenous variables. The results indicated that perceived need-supportive coaching behavior positively predicted subjective task value (*β* = 0.286, *p* < 0.001) and task orientation (*β* = 0.217, *p* < 0.001). In turn, subjective task value (*β* = 0.588, *p* < 0.001) and task orientation (*β* = 0.323, *p* < 0.001) positively predicted athlete engagement. Additionally, subjective task value positively predicted task orientation (*β* = 0.391, *p* < 0.001), and need-supportive coaching behavior had a significant positive effect on athlete engagement (*β* = 0.158, *p* < 0.01). Moreover, the squared multiple correlation (*R*^2^) for athlete engagement was 0.734, indicating that need-supportive coaching behavior, subjective task value, and task orientation together explained 73.4% of the variance in athlete engagement, demonstrating that the proposed research model has strong statistical power in explaining athlete engagement.

Finally, to validate the proposed hypotheses, this study evaluated the direct and indirect effects of need-supportive coaching behavior on athlete engagement. As shown in Table 3, need-supportive coaching behavior had significant direct (*β* = 0.121, *Z* = 3.667, *p* < 0.001) and indirect effects (*β* = 0.209, *Z* = 5.500, *p* < 0.001) on athlete engagement, indicating that the structural model is partially mediated. Further specific indirect effects indicated that need-supportive coaching behavior positively predicted athlete engagement through subjective task value (*β* = 0.128, *Z* = 4.000, *p* < 0.001) or task orientation (*β* = 0.053, *Z* = 3.118, *p* < 0.001). Meanwhile, subjective task value and task orientation sequentially mediated the relationship between need-supportive coaching behavior and athlete engagement (*β* = 0.028, *Z* = 3.500, *p* < 0.001). Moreover, the results from 5,000 bootstrap samples showed that their confidence intervals did not contain zero, providing further evidence for the direct and indirect effects of need-supportive coaching behavior on athlete engagement. Additionally, this study compared these three specific indirect effects and found that perceived task value was more important than the other two factors (see Table 3).

**Table 3:** Direct, indirect, and total effects of the statistical model.

Relationships	Point estimation	Product of coefficients		Bootstrapping			
				BC 95% CI		Percentile 95% CI	
		SE	*Z*	Lower	Upper	Lower	Upper
Specific indirect effects							
NSCB→STV → AE	0.128	0.032	4.000	0.071	0.194	0.069	0.191
NSCB→TGO → AE	0.053	0.017	3.118	0.025	0.093	0.023	0.090
NSCB→STV → TGO → AE	0.028	0.008	3.500	0.015	0.049	0.013	0.046
Contrasts							
STV-TGO vs. STV	−0.100	0.029	−3.448	−0.166	−0.052	−0.162	−0.049
STV-TGO vs. TGO	−0.026	0.019	−1.368	−0.065	0.011	−0.065	0.010
TGO vs. STV	0.075	0.039	1.923	0.001	0.155	0.000	0.153
Total indirect effect	0.209	0.038	5.500	0.140	0.286	0.137	0.284
Direct effect	0.121	0.033	3.667	0.059	0.190	0.057	0.188
Total effect	0.330	0.045	7.333	0.247	0.425	0.244	0.422

*N = 379. 5,000 bootstrap sample; SE, standard error; BC, bias corrected; CI, confidence interval; NSCB, need-supportive coaching behavior; TGO, task orientation; STV, subjective task value; AE, athlete engagement.*

## 5 Discussion

This study investigated relationships between perceived need-supportive coaching behaviors, subjective task value, task orientation, and engagement in sports among Chinese high school football players. Based on relevant theories and research advancements, this study hypothesized that need-supportive coaching behavior would have a direct effect on athlete engagement and that subjective task value and task orientation would mediate the relationship between need-supportive coaching behavior and athlete engagement. The SEM analysis results supported the proposed multivariate sequential mediation model. Following this, an in-depth discussion and further interpretation of the study results was conducted, taking into consideration the literature review and related theories.

The first objective of the study was to determine the relationship between need-supportive coaching behavior and athletes’ engagement. It was clear that the findings from the analysis supported hypothesis 1, that need-supportive coaching behavior is positively related to athlete engagement (*β* = 0.121, *Z* = 3.667, *p* < 0.001). This finding supports the assumption of self-determination theory and is consistent with that of De Muynck et al. (2020), Gao et al. (2021), and Mellano et al. (2020). The study results can be interpreted from the perspectives of autonomy, competence, and relatedness support. Although this study only investigated one general dimension of need-supportive coaching behavior, the SEM analysis also seemed to provide potential support for this interpretation. Specifically, coaches’ autonomy support offers a potential strategy for positively predicting athletes’ three basic needs and promoting their engagement in sports (Pedro and Martins, 2017; Jowett et al., 2020). Also, studies have shown the importance of competence support in fostering and maintaining individuals’ motivation and participation in sports (Curran et al., 2016; de et al., 2020; Podlog et al., 2015). Moreover, coaches’ relatedness support can enhance athletes’ effort, persistence, and engagement (Gonzalez-Peno et al., 2021; Guo et al., 2021; McGee and DeFreese, 2019). Additionally, there was a moderate correlation between need-supportive coaching behavior and athlete engagement (*r* = 0.432, *p* < 0.01), while the path coefficient (*β* = 0.121, *p* < 0.01) was relatively small, indicating a greater extent of mediation between them. This suggests that need-supportive coaching behavior not only directly predicts athlete engagement but also indirectly predicts their engagement in sports by fostering intrinsic motivation.

The second objective of this study discussed how need-supportive coaching behavior indirectly influences athletes’ engagement through subjective task value. The results showed that athletes’ perceived task value has a specific mediating effect between need-supportive coaching behavior and athlete engagement (*β* = 0.128, *Z* = 4.000, *p* < 0.001), which supports hypothesis 2. The literature in physical education has also reported that the need-supportive behaviors of significant others can determine individuals’ perceived task value, which in turn promotes their persistence, effort, and engagement in physical education (Olivier et al., 2020; Shang et al., 2022; Zhang et al., 2012). One possible explanation is that the autonomy-supportive, encouraging, and caring atmosphere provided by coaches may lead athletes to perceive the intrinsic importance, usefulness, and enjoyment of the current task. For instance, previous studies have found that individuals’ perceived caring climate (Jung, 2019) or structured curriculums (Pang, 2014) positively impact their perceived task value. Additionally, several studies have found that people tend to be involved in things they find important, personally meaningful, and interesting, whether in sports or other domains (Wang et al., 2017; Wigfield and Eccles, 2020; Selänne et al., 2016). As a result, a supportive motivational style creates a nurturing pattern for adaptive motivation and engagement among athletes, which enhances their perceived task value. In turn, the perceived importance, usefulness, and interest of the task further promote their engagement in sports.

The third objective of this study discussed how need-supportive coaching behavior indirectly influences athletes’ engagement through task orientation. Data analysis revealed that athletes’ task orientation mediated the relationship between need-supportive coaching behavior and athlete engagement (*β* = 0.053, *Z* = 3.118, *p* < 0.001), supporting hypothesis 3. The basic psychological needs support from coaches can positively predict athletes’ task orientation, which in turn influences their engagement in sports. One possible explanation is that coaches’ autonomy, competence, and relatedness support motivate athletes to focus more on mastering knowledge or skills (Shih, 2013; Diseth and Samdal, 2014; Iwasaki and Fry, 2016; Nicholls et al., 2017). Furthermore, achievement goal theory and its related research advances also suggest that individuals’ task orientation can positively predict athlete engagement (Atkins et al., 2015; Ruiz-González et al., 2015; Sun et al., 2015; Iwasaki and Fry, 2016; Lodewyk, 2019; D’Astous et al., 2020). Overall, the study findings confirm the mediation effect of task orientation in the relationship between need-supportive coaching behavior and Chinese high school football players’ engagement.

The fourth objective of this study discussed the sequential mediating effect of subjective task value and task orientation between need-supportive coaching behavior and athletes’ engagement. The results showed that athletes’ task value perception and task orientation had a significant sequential mediating effect between need-supportive coaching behavior and athlete engagement (*β* = 0.028, *Z* = 3.500, *p* < 0.001), supporting hypothesis 4. This study confirms previous findings, revealing that need-supportive coaching behavior can influence their task orientation through task value perception, which in turn affects their engagement in sports (Wentzel et al., 2017; Von Keyserlingk et al., 2022). In other words, the need-supportive environment created by coaches not only fosters athletes’ intrinsic, attainment, and utility value perceptions of tasks but also encourages them to focus more on mastering task skills, thereby determining their level of engagement in training or competitions. Taken together, need-supportive coaching behavior can directly predict athlete engagement and indirectly influence athlete engagement via the sequential mediation effects of subjective task value and task orientation. Although this study found that subjective task value might be a more important factor, the other two mediating effects also showed significant positive effects. Therefore, it is recommended that coaches fully utilize need-supportive coaching styles during training or competitions, emphasize cultivating athletes’ perception of task value, and actively guide athletes toward a task-oriented goal to enhance their engagement in training and competitions.

Finally, it is worth noting that this study focuses on Chinese high school football players, but athletes of different age groups may respond differently to coaches’ need-supportive behaviors. For example, younger athletes may rely more on direct guidance from coaches and exhibit relatively weaker autonomy needs, whereas older athletes tend to respond more positively to autonomy-supportive behaviors (Deci and Ryan, 2000; Reeve, 2009). Additionally, athletes with varying talent levels and personality traits may interpret and react to need-supportive behaviors differently. For instance, highly talented players may require more autonomy to align with their self-development needs, thereby further enhancing their engagement. On the other hand, less talented players may need more emphasis on fundamental skill development and motivational guidance to improve their perceived value of sports tasks, ultimately increasing their engagement (Croft et al., 2023). Furthermore, cultural differences significantly affect the interaction and engagement between coaches and athletes. As Nakamura (2024) found, a lack of cultural sensitivity in cross-cultural teams may lead to misunderstandings of coaching behaviors, reducing athletes’ engagement. Therefore, the generalization of research findings must fully consider these factors and their impact on the mechanisms of coaching behaviors.

### 5.1 Contributions and implications

This study reveals that the perceived need-supportive coaching behaviors not only directly influence athlete engagement, but also influence their engagement in sports through subjective task value or task goal orientation, as well as their sequential mediating role. This study has practical significance for Chinese campus football training practices and athlete motivations and engagement. As Ryan and Deci (2018) mentioned, “coaching can be a strong asset in both personal development and organizational performance.” Therefore, in training and competitions, coaches should actively create a motivational climate that supports autonomy, competence, and relatedness. This includes giving athletes more freedom of choice, implementing structured organizational strategies, and providing sufficient care and support. These factors are key determinants of athletes’ task value perception and their inclination toward task-oriented goals, which will enhance their engagement in sports. For football organizations, educational and government agencies, the findings of this study offer additional actionable insights. For example, football organizations can create a culture that prioritizes the motivational strategies of coaches and the goals and values of athletes. Additionally, nurturing collaboration between coaches can facilitate the sharing of best practices and enhance the overall coaching environment. Educational and government agencies could fund longitudinal research and organize training workshops for coaches, aimed at disseminating effective need-supportive practices across diverse sports settings. Taken together, the findings provide additional practical implications for various stakeholders, and these efforts are conducive to the popularization and development of campus football in China.

### 5.2 Limitations and future research

This study had limitations in several aspects. First, the cross-sectional design employed lacked longitudinal data, making it impossible to accurately infer causal relationships. Future research could consider using a longitudinal design to explore the relationships between these constructs. Second, the study sample was drawn from only one region in China, limiting generalizability. Further studies should examine a broader sample range, encompassing athletes from different regions and competitive levels. Moreover, data was collected through athletes’ self-reports, possibly leading to measurement bias. Future studies could integrate coach evaluations and other objective indicators to further enhance the accuracy of the data. Furthermore, there may be some degree of overlap between the conceptual dimensions and operational definitions of athlete engagement and some measures of subjective task value and task orientation. Future research could use both qualitative and quantitative techniques to better understand the multidimensional nature of athlete engagement and reduce the overlap between different constructs. Lastly, further exploration of the impact of different coaching styles on athletes’ subjective task value, task orientation, and engagement would have provided valuable insights for the research. Taken together, future studies should address these limitations and delve deeper into the mechanisms and influencing factors of athlete engagement, thereby offering more valuable theoretical and practical guidance for promoting sports participation and development.

## 6 Conclusion

This study found that need-supportive coaching behavior was positively related to athlete engagement. Further analysis revealed that subjective task value and task orientation serve as significant independent and sequential mediators between need-supportive coaching behavior and athlete engagement. It is evident that need-supportive coaching behavior plays a crucial role in athletes’ motivation and engagement. Based on these conclusions, coaches should actively create a need-supportive coaching environment, such as giving athletes freedom of choice, offering structured guidance and positive feedback, and establishing effective interaction mechanisms. This need-supportive coaching style is a key factor in athletes’ motivation and engagement. Overall, the study findings not only contribute a novel perspective to understanding athlete engagement mechanisms but also offer practical guidance for coaching practices in Chinese campus football.

## Data Availability

The raw data supporting the conclusions of this article will be made available by the authors, without undue reservation.
